# Spatio-temporal patterning of extensile active stresses in microtubule-based active fluids

**DOI:** 10.1093/pnasnexus/pgad130

**Published:** 2023-04-12

**Authors:** Linnea M Lemma, Minu Varghese, Tyler D Ross, Matt Thomson, Aparna Baskaran, Zvonimir Dogic

**Affiliations:** Department of Physics, Brandeis University, 415 South St., Waltham, 02453 MA, USA; Department of Physics, University of California, Santa Barbara, 93106 CA, USA; Department of Physics, Brandeis University, 415 South St., Waltham, 02453 MA, USA; Department of Computing and Mathematical Sciences, California Institute of Technology, 1200 E California Blvd. Pasadena, 91125 CA, USA; Division of Biology and Biological Engineering, California Institute of Technology, 1200 E California Blvd., Pasadena, 91125 CA, USA; Department of Physics, Brandeis University, 415 South St., Waltham, 02453 MA, USA; Department of Physics, University of California, Santa Barbara, 93106 CA, USA

**Keywords:** active matter, molecular motors, fluid dynamics, optical control

## Abstract

Microtubule-based active fluids exhibit turbulent-like autonomous flows, which are driven by the molecular motor powered motion of filamentous constituents. Controlling active stresses in space and time is an essential prerequisite for controlling the intrinsically chaotic dynamics of extensile active fluids. We design single-headed kinesin molecular motors that exhibit optically enhanced clustering and thus enable precise and repeatable spatial and temporal control of extensile active stresses. Such motors enable rapid, reversible switching between flowing and quiescent states. In turn, spatio-temporal patterning of the active stress controls the evolution of the ubiquitous bend instability of extensile active fluids and determines its critical length dependence. Combining optically controlled clusters with conventional kinesin motors enables one-time switching from contractile to extensile active stresses. These results open a path towards real-time control of the autonomous flows generated by active fluids.

**Significance Statement**

Being driven away from equilibrium by microscopic force-generating processes, the cellular cytoskeleton enables life-sustaining functions such as cytoplasmic streaming, cell motility, and cell division. Active fluids reconstituted from well-characterized biochemical components have the potential to replicate such lifelike phenomena in synthetic materials. However, in biological cells, mechanical functions are enabled by force generation that is under tight spatio-temporal control. In comparison, cytoskeletal active matter is fueled by microscopic activity that is generated uniformly throughout a sample. Controlling force-generating patterns is the key to building more complex materials and functions and mimicking biology. Motivated by these observations we develop and characterize optically responsive molecular motors that allow for robust and repeatable spatio-temporal patterning of active flows in microtubule-based active matter.

## Introduction

The shapes of cells, tissues, and organs are determined by the patterns of the mesoscopic active stresses, which are collectively generated by molecular motors (1–6). Developing analogous synthetic force-generating materials is of fundamental interest and is essential for diverse applications (7), ranging from microfluidics and adaptive optics to soft robotics. Active matter, which is an assemblage of microscopic force-generating constituents, provides a promising experimental platform for realizing these goals (8). Bulk active materials exhibit diverse dynamical states (9–14), which can exhibit chaotic or turbulent-like flows. However, developing soft active matter systems is only the first step towards creating life-like synthetic materials; one also needs to harness their force-generating capabilities to stabilize a targeted dynamical state. In this vein, previous work demonstrated that immutable geometrical confinement can stabilize spinning vortices or long-ranged coherent flows (15–18).

The next challenge is to develop protocols that control transitions between distinct dynamical states *in situ*. For inspiration, one can look towards biological cells, which can sense their environment and adjust force patterns accordingly (19). A first step for achieving analogous capabilities in synthetic materials is to develop active matter whose dynamics are responsive to external cues. Light patterns have been used to control motility induced phase separation and the structure and dynamics of bacteria (12, 13, 20–23). Recent advances also demonstrated optical control of cytoskeletal active matter, including the control of local contractility and aster formation, as well as influencing the motion of topological defects (24–26). Here we demonstrate robust and repeatable spatial and temporal control of active stresses in three-dimensional microtubule-based active fluid. To achieve precise control, we build upon opto-kinesin (24) to develop a new single-headed kinesin motor that exhibits optically induced enhanced clustering. The unique features of this system enable temporal control of the ubiquitous bend instability of active fluids, and reveal how this instability depends on the system size.

### Optical control of kinesin clustering

Conventional microtubule-based active fluids are powered by clusters of kinesin-1 molecular motors (9). Motors within a single cluster bind adjacent microtubules. Depending on the microtubules’ relative polarity, the motor clusters induce interfilament sliding and bundle extension (27). At high microtubule concentrations, extensile bundles form a percolating network that is continuously reconfigured by the repeating cycles of motor-driven bundles extending, buckling, fragmenting, and re-annealing (11). Such network dynamics drive the turbulent-like flows of the background fluid. Coupling energy-efficient kinesin motors with an ATP regeneration can sustain non-equilibrium dynamics for hours or even days (28).

Typically, microtubule-based active matter is powered by a 401 amino acid fragment of the *Drosophila* kinesin-1 motor (9, 11). For these motors, two fragments associate to form a dimeric kinesin motors, which we refer to as K401. These processive motors are continuously bound to a microtubule as they take consecutive steps (29, 30). K401 motors are labeled with a biotin. Adding streptavadin induces the irreversible assembly of higher order clusters containing several K401 motors, which efficiently power active matter dynamics.

Microtubule-based active matter can also be powered by a monomeric 365 amino-acid fragment of *Drosophila* kinesin-1 motor, which we refer to as K365 (28, 31). Having a shorter neck linker domain, such fragments do not dimerize. K365 motors are not processive; they detach from a microtubule after each step (32, 33). Similar to K401, K365 kinesin motors are labeled with biotin and can assemble into higher order streptavidin clusters. Notably, the K365 motor clusters can bind multiple filaments and induce their relative sliding, generating active stresses. The dynamics of an active fluid driven by clusters of K365 motors exhibit greater temporal stability of flow speeds and structural length scales, when compared to those driven by K401 motors (28). Clustering can transform non-processive motors into processive ones (34).

Multimotor clusters are essential for generating active stresses. Isolated motors move along separate filaments, but are unable to generate interfilament sliding. The biotin-streptavidin interaction used to assemble conventional clusters is an essentially irreversible non-covalent bond (35). Decreasing intra-cluster bond strength, decreased the efficiency of interfilament sliding, as was revealed by DNA-based clusters, in which the binding strength can be precisely tuned (31). Below a minimal bond strength, the clusters were unable to generate active stresses.

**Fig. 1.**
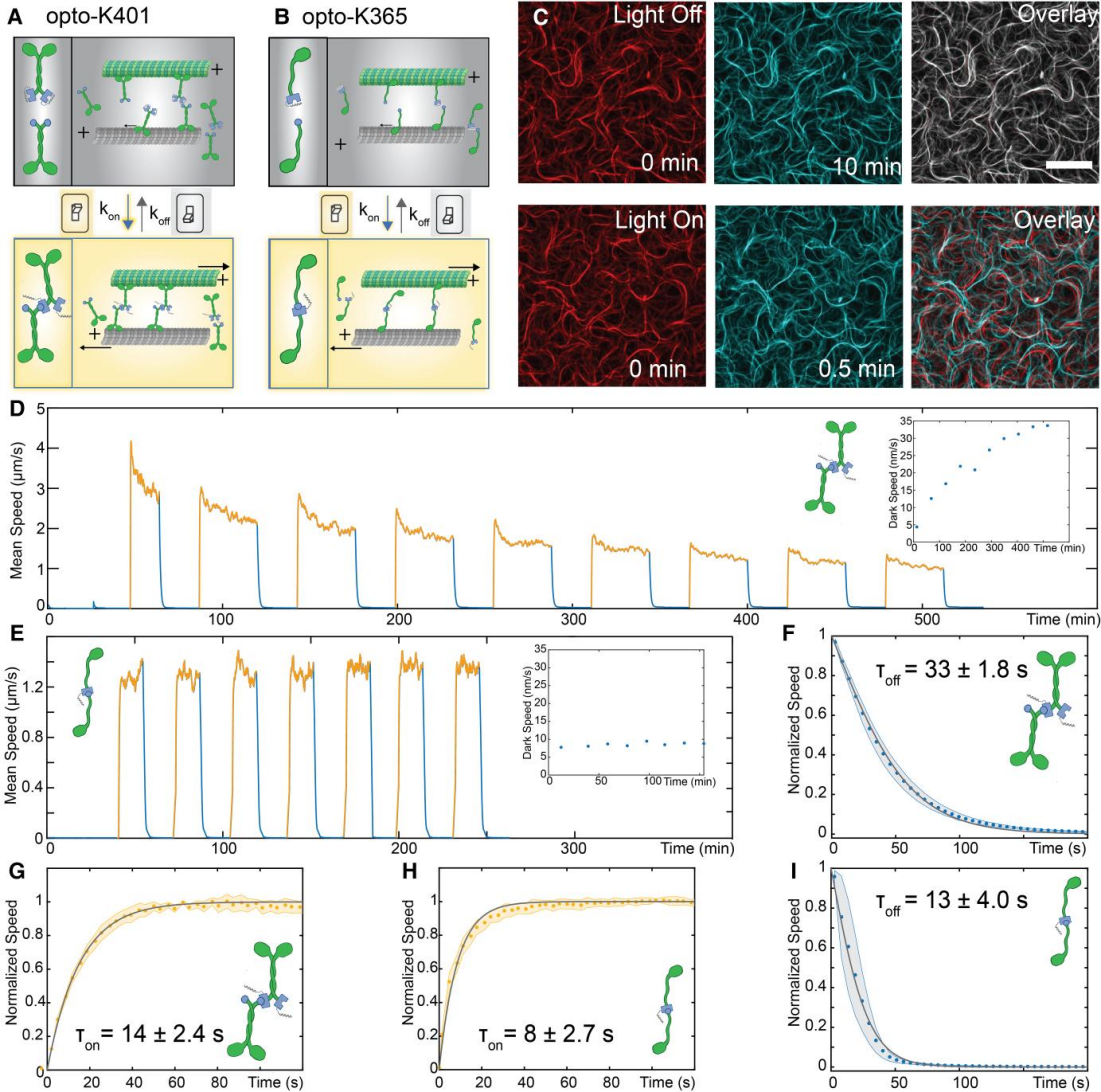
Optical switching between an active extensile fluid and an inactive solid. A) The iLID optogenetic domains are fused to processive kinesin motors. When the protein is illuminated with blue light, the iLID domain changes conformation and the complementary constructs bind to each other. The clustered motors generate interfilament sliding. B) The iLID domains are fused to a K365 truncated kinesin motor. The K365 motor clusters also dimerize and induce interfilament sliding when exposed to blue light. Schematics of microtubules and kinesin motors were made using BioRender.com. C) An active network of labeled microtubules powered by optogenetic kinesin clusters, K365-opto. Overlaid initial (red) and final frame (cyan) show the network dynamics (red + cyan = gray). top: In the dark, the microtubule bundles do not move. bottom: When exposed to blue light, the bundles extend and buckle generating active flows. K401-opto driven dynamics are qualitatively the same (Video 1). Scale bar, 25 μm. D) Speed of active flows generated by processive opto-kinesin clusters. Activation is cycled off (blue) and on (gold). In the on-state, the light is pulsed for 10 ms every 5 s. Inset: the average network speed in the dark state with subsequent photo-activation cycles. E) Active flow speeds with K365 opto-kinesin. Inset: dark speed over time. F) The decay of the flow speed upon turning the light off for processive motors. The shaded region is the standard deviation (n=9). The line is a fit to Eq. 2. G) For processive motors, the increase in speed upon photoactivation. The line is a fit to Eq. 1. H) The increase in speed after photoactivation for K365 (n=7). I) The decay in the flow speed after deactivation for K365 motors.

Such considerations suggest that controlling cluster assembly provides a route towards control of active stresses. Indeed, a recent advance fused optically responsive dimerizing domains (improved light-induced dimers, iLID) to kinesin motors to establish reversible light control of the assembly of the kinesin clusters (24, 36). In the absence of photoactivation, the binding affinity of the kinesin-iLID to its polypeptide binding partner, which is attached to another kinesin motor, is low: thus the kinesin motors are largely isolated. They consume ATP and walk along microtubules, but do not induce significant interfilament sliding [Fig. 1A and B]. When photoactivated, the iLID domain changes conformation, exposing the binding site and increasing the affinity for motor pairs to dimerize. The newly formed motor clusters can cross-link and slide microtubules [Fig. 1A and B]. Such opto-kinesin clusters can induce photoactivated contraction of microtubule asters and generate programmable fluid flows (24).

## Results

### Steady-state dynamics of active fluids powered by opto-kinesin clusters

We first explored the ability of K401-opto clusters to control dynamics of microtubule-based extensile fluids [Fig. 1A]. In the presence of blue light, opto-clusters generated extending bundles that continuously buckled and annealed [Video 1, Supplmentary Material: Materials and Methods]. Images taken 30 s apart revealed a continuously reconfiguring microtubule network [Fig. 1C, bottom]. Upon deactivation, the microtubule bundles quickly stopped extending and remained frozen in their paused configuration. Images taken 10 min apart demonstrate the lack of measurable dynamics in the absence of optical signal [Fig. 1C, top]. When exposed to continuous blue light, the photoactivated flows decayed rapidly, perhaps due to irreversible cross-linking of motors clusters. Pulsed 10 ms photoactivation every 5 s avoided this issue. Additionally, we optimized the motor concentration to achieve maximal difference between the dark and photoactivated state [Supplementary material: Materials and Methods].

We measured the speed of the microtubule-powered flows through multiple cycles of photo-activation, using particle image velocimetry (PIV) of the fluorescent microtubule images (37) [Fig. 1D, Fig. S1, Materials and Methods]. After formation of the bundled network, the sample was alternately exposed to dark and light cycles each lasting 15 min and 1 h, respectively. When illuminated active flows increased 100-fold from ∼20 nm/s in the dark to ∼2 μm/s in the light. The difference in speeds persisted over several light cycles that spanned the active network lifetime.

Quantifying the flow speeds, however, revealed subtle variations of photoactivated dynamics over time. First, within a single cycle, the maximum speed was reached within 30 s after photoactivation and then decayed to a plateau. The decay timescale increased with subsequent photo-activation cycles. Second, the plateau speed decreased with each successive photo-activation cycle. Such decay is reminiscent of aging observed in active fluids driven by the conventional K401 motor clusters (28). Finally, the background speed in the dark state increased throughout the sample lifetime [Fig 1D inset]. The combination of increased dark flows and decreased photoactivated flows reduced the contrast between the dark and the illuminated states [Fig. S2]. These behaviors present a challenge for applications that require repeatable control of the active stress.

Motivated by the observation that K365 kinesin clusters generate more regular temporal dynamics (28), we created clusters which fused a monomeric kinesin fragment (K365) to both iLID domains [Fig. 1B, Fig. S3]. The K365 opto-clusters also powered the extensile dynamics upon photo-activation [Video 2]. After formation of the bundled network, the active fluid was exposed to repeating cycles of photo-activation. In contrast to the K401-opto motors, the K365-opto constructs exhibited a regular, reversible, and reproducible response. Photoactivated flows immediately plateaued, at 1.2 μm/s, while dark state flows were ∼7 nm/s. Subsequent photo-activation cycles generated the same speed, with no significant change over the sample lifetime. Finally, the dark state flow speeds did not increase over time, and were effectively zero when compared to enzymatically dead samples [Fig. 1E inset, Fig. S4]. Thus, K365 opto-kinesin clusters allow for robust and repeatable spatio-temporal control of active stresses.

The steady-state dynamics of extensile fluids is uniquely suited to characterize the temporal response of clusters to optical stimulus, which is related to the kinetics of the kinesin cluster assembly and disassembly. We measured the timescale for which the flows reached their maximum speed after photo-activation. For both K401-opto and K365-opto motor clusters, the increase in speed was fitted to a bounded exponential function


(1) $$\langle |v(t)|\rangle /v_{\max}=1-e^{-t/\tau_{\mathrm{on}}}.$$

For K401 clusters, $\tau_{\mathrm{on}}=14\pm 2.4$ s and for K365 clusters $\tau_{\mathrm{on}}=8\pm 2.7$ s [Fig. 1G and H]. Similarly, we measured the time scales for the flows to cease after deactivation. We fitted the velocity decays to a logistic function:


(2) $$\langle |v(t)|\rangle =\frac{2}{1+e^{-t/\tau_{\mathrm{off}}}},$$

from which we extracted $\tau_{\mathrm{off}}=33\pm 1.8$ s for K401 clusters and $\tau_{\mathrm{off}}=13\pm 4.0$ s for K365 clusters [Fig. 1F and I]. The relative timescales of the two motors were independent of the functional forms of Eqs. 1 and 2.

### Controlling the speed of extensile flows

**Fig. 2.**
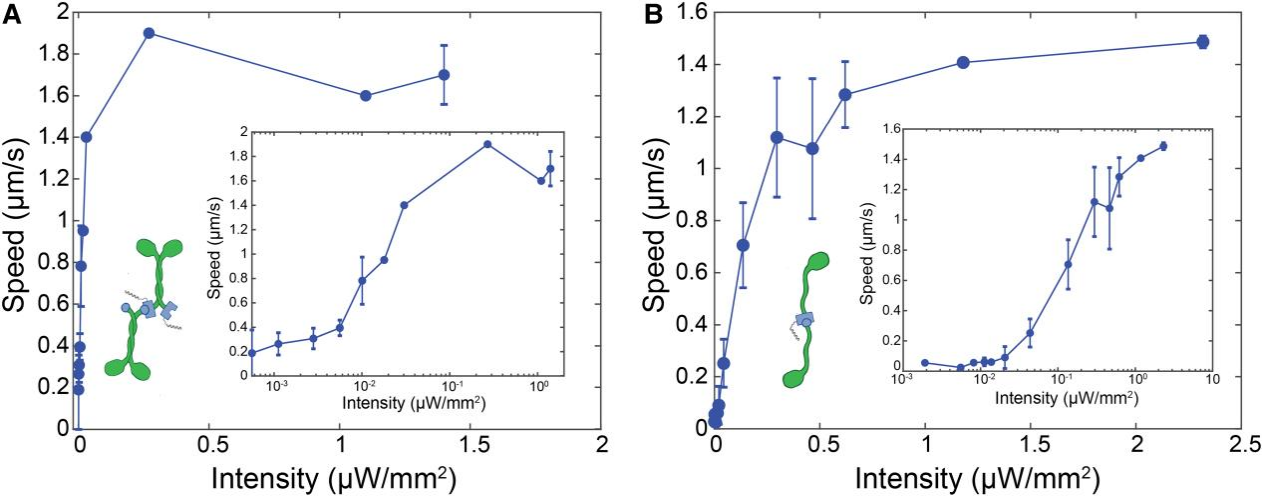
Signal intensity controls speed of active flows. A) Autonomous flow speed as a function of the light intensity for processive motors (n=2, 2, 2, 2, 2, 2, 1, 1, 1, 3 Intensity = 0 → 2 μW/mm${}^{2}$). B) Speed of autonomous flows measured through PIV versus the light intensity for K365 motors (n=3, 2, 4, 3, 1, 2, 3, 4, 2, 3, 2, 2, 2, Intensity = 0 → 2.5 μW/mm${}^{2}$). Insets: Log-log scaling of axes. Error bars represent standard error on multiple samples’ flows.

We next aimed to control the speed of active flows. Absorbance assays of AsLOV proteins, the native protein from which iLID was derived, indicated that the light intensity controls the fraction of dimerized domains (38). Thus, we expect that the light intensity also controls the concentration of bound motor clusters, which in turn controls the magnitude of the active stress and the flow speeds. We measured the dependence of the autonomous flow speeds on the intensity of 488 nm illuminating light at the sample plane [Fig. 2, Supplementary material: Experimental Methods]. With increasing light intensity, the flow speed increased until 0.4 μW/mm${}^{2}$ for K401-opto [Fig. 2A, Fig. S5]. The K365-opto motors exhibited increasing flow speeds with increasing light intensity until saturating at 1 μW/mm${}^{2}$ [Fig. 2B]. Below 0.01 μW/mm${}^{2}$, the measured flows asymptotically approached the dark state [Fig. 2A and B inset]. The K401-opto clusters achieved a higher saturating speed, at a lower light intensity than the K365-opto clusters. Additionally, K401-opto motor clusters exhibit a speed of ∼0.2 μm/s as intensity approaches zero, indicating non-specific binding of motors. In contrast, the speed driven by K365-opto motor clusters approaches zero as intensity approached zero.

### Controlling the extensile bend instability

**Fig. 3.**
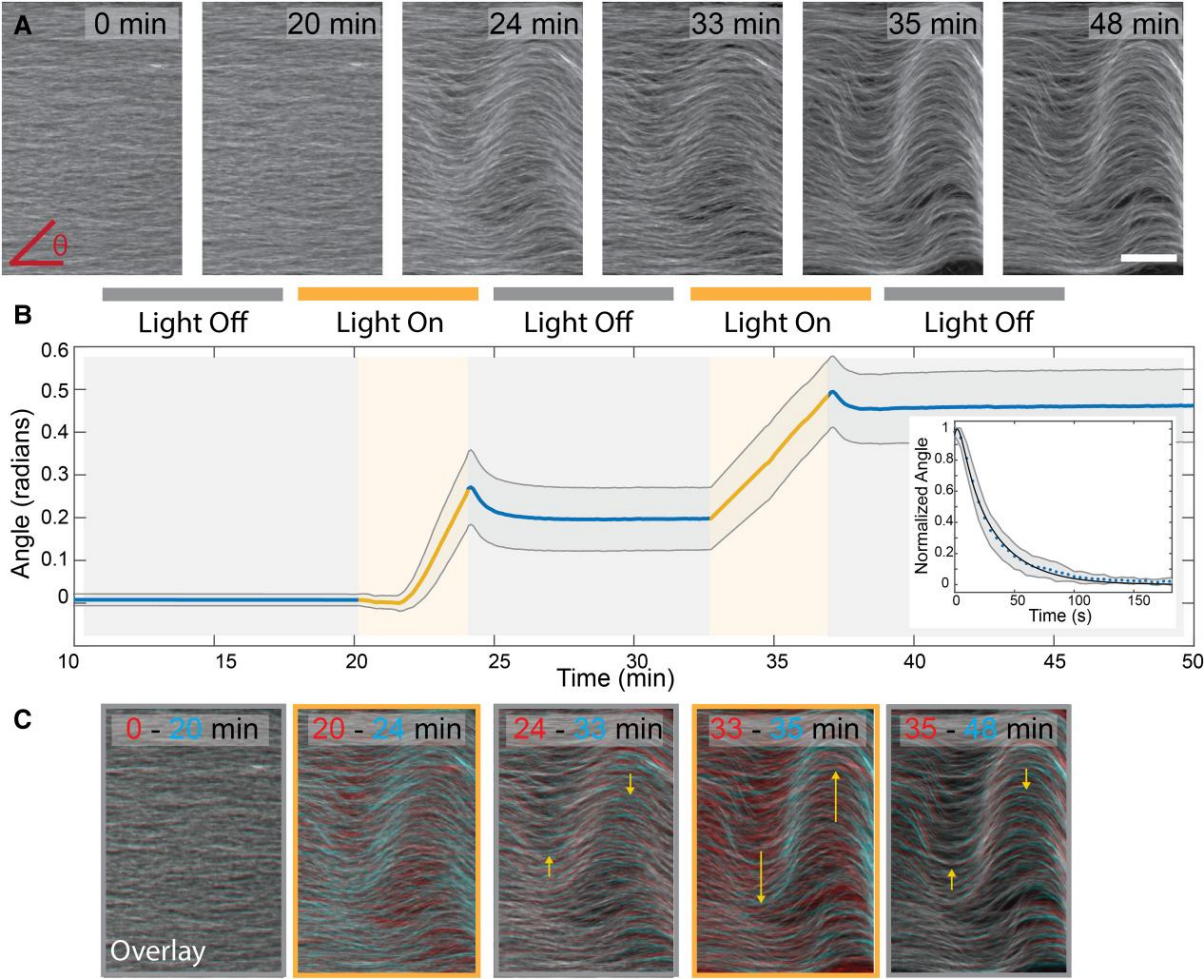
Controlling the bend instability. A) Time series of a flow aligned opto-kinesin microtubule network exposed to alternating dark and light conditions. Scale bar, 200 μm. B) The time-evolution of the average in-plane angle of deformation for two photo-activation cycles. Inset: The average in-plane angle as a function of time elapsed after deactivation. The shaded region is standard deviation (n=5). C) Overlays of time points in (a) with early time in red shows relative microtubule movement across each light cycle. Yellow arrows direct the eye forward in time.

To demonstrate temporal control of active stresses, we used K365 opto-clusters to probe the bend instability of shear-aligned microtubules, which is a ubiquitous feature of extensile active fluids (39–42). Once aligned due to shear flow, microtubules remained quiescent in the absence of light [Fig. 3A]. When exposed to blue light, the opto-kinesin clusters generated extensile sliding, which in turn powered the growth of the bend instability. Upon deactivation, the bend instability stopped [Fig. 3A, Video 3]. The individual microtubule bundles within the deactivated network straightened slightly, but the material only partially relaxed towards its initial uniformly aligned state. Similar deformation dynamics occurred over multiple cycles of photo-activation, with motor clusters pushing the microtubules into an active bend, and the network partially relaxing when deactivated.

To quantify the bend instability, we measured the average in-plane filament angle ⟨θ⟩ away from the initial alignment, by calculating the local structure tensor from fluorescence images [Fig. 3B, Fig. S6]. In this analysis, we treat the system as quasi-2D since the bend instability was predominantly in-plane. During the photo-activation, the average angle ⟨θ⟩ grew as the bend instability developed. Upon deactivation, the average angle decayed, but after several minutes attained a finite-value plateau. Such dynamics are seen by overlaying the initial and final images upon photoactivation [Fig. 3C].

The decay in the average angle upon deactivation was described by:


(3) ⟨θ(t)⟩=M(t)A(t)+R(t).

**Fig. 4.**
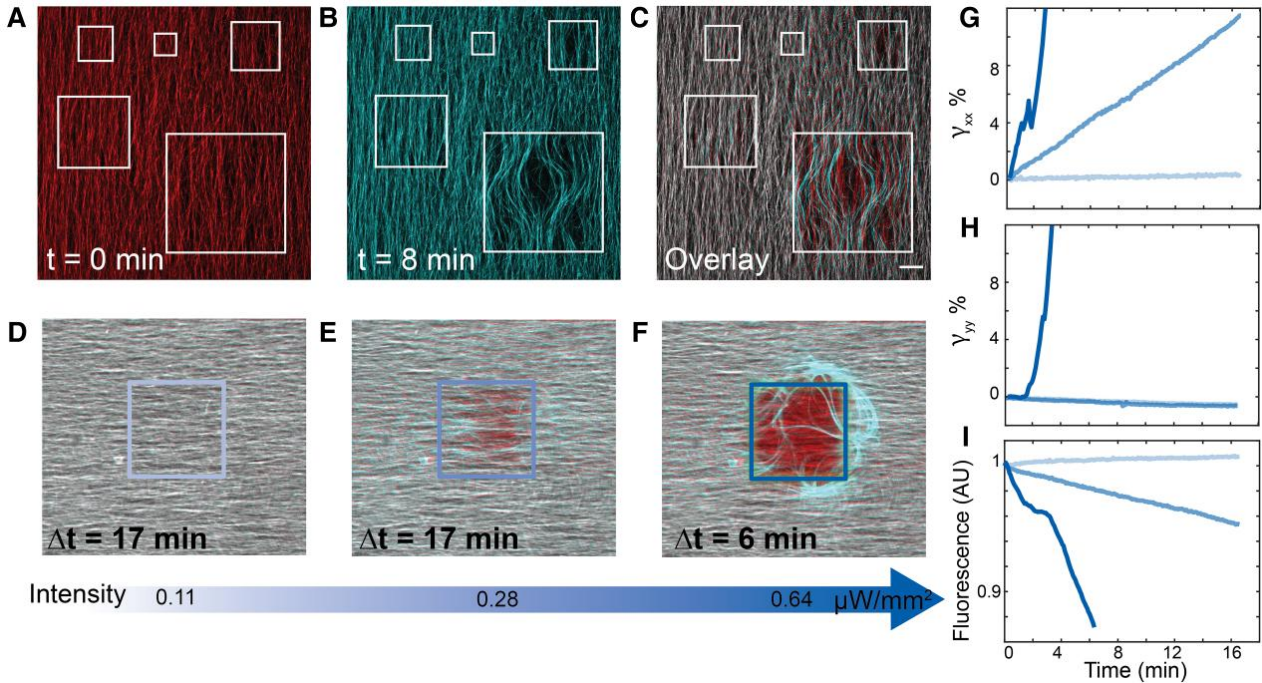
Instability of confined active fluid. A) White squares indicate activation regions from 50 μm${}^{2}$ to 500 μm${}^{2}$ of aligned microtubules. B) Microtubule network after 8 min of constant photoactivation within the confined regions. C) The overlay of the initial and final images. D–F) The dynamics of the quiescent, sliding and buckling regime is illustrated by overlaying of initial (red) and final (cyan) state of a flow aligned network. 400×400μm square region was activated. Gray indicates no movement of filaments (red + cyan = gray). G) $\gamma_{xx}$ within the activated region over time for each activation intensity. H) $\gamma_{yy}$ within the activated region over time for the three regimes. I) The average fluorescence intensity inside the activated region over time for the three regimes.

Here, M(t) describes the unbinding kinetics of the motor clusters, A(t) describes the activity-driven angle growth and R(t) describes the relaxation of the passive microtubule network. The activity drives linear growth such that $A(t)=\dot{\gamma }t$, where $\dot{\gamma }=0.002$ rad/s is the measured angular growth rate [Fig. S7]. Upon deactivation, growth slowed due to unbinding of opto-clusters. To account for this effect, we multiply the growth rate by the rate at which the clusters fall apart. Studies of unbinding kinetics of the light activated domain revealed exponential decay, $M(t)=e^{-t/\tau }$ where τ = 24 s [Fig. S8, Supplementary material: Elastic Relaxation] (43). Modeling the microtubule network as an elastic solid, predicts $R(t)=e^{-t/r}$ where *r* is the elastic relaxation time. Thus:


(4) $$\langle \theta \rangle =e^{-t/\tau }\dot{\gamma }t+Ce^{-t/r}$$

where τ is the characteristic unbinding time for the motors and *C* is a scaling fit parameter. To fit the data, we fixed $\dot{\gamma }=0.002$ rad/s, and left the time scales τ and *r* as free parameters [Fig. 4B inset, Fig. S9]. Experimental fits yielded τ=17±5.5 s which is within error of the previously reported unbinding kinetics of the iLID domain (43). We also extracted, the elastic relaxation time r=17±9.8 s.

### Spatial patterning of active stresses

Theory predicts that the bend instability only develops when the sample size is larger than a critical length scale, which in turn is determined by the ratio of the sample elasticity to the activity (39, 44). Opto-kinesin clusters enable application of spatially patterned stress, which can elucidate how the interplay between geometry and activity controls the onset of the bend instability.

Shear flow associated with chamber loading induced initial alignment of microtubule bundles. When uniformly illuminated, such samples exhibited the previously described bend instability [Fig. 3] (42). We used a laser scanning confocal microscope to photoactivate square regions ranging in size from 50×50μm${}^{2}$ to 500×500μm${}^{2}$. At a single photoactivation intensity, the 500×500μm${}^{2}$ region exhibited the bend instability, while the smaller regions remained quiescent, demonstrating a size-dependent instability [Fig. 4A to C, Video 4].

To quantify the size-dependent instability, we systematically changed the photoactivation intensity within a single region. We placed an opaque mask above the sample with a 400×400μm${}^{2}$ square opening. Exposing such samples to light photoactivated the exposed area, while leaving the rest of the sample inactive. With sequentially increasing light intensity, we observed three regimes [Video 5]. At very low intensities, the microtubules inside the activated region remained stationary [Fig. 4D]. Beyond a threshold intensity, the microtubules started sliding past each other along the alignment direction, but there was no deformation in the perpendicular direction [Fig. 4E]. Finally, at high intensities, the microtubules buckled in the direction perpendicular to alignment [Fig. 4F]. We refer to these as the quiescent, sliding, and buckling regimes.

To quantify these observations, we measured the microtubule displacement field after the region was photoactivated for a defined time [Fig. S10]. The direction of the initial alignment was the *x*-axis, while *y*-axis was the perpendicular direction in the image plane. In-plane deformations dominated the instability at these confinements, as long as the microtubules were initially well aligned. Thus, for the purposes of this analysis, we neglected the out of plane component. We calculated the build up of strain over time by averaging $\gamma_{xx}$ and $\gamma_{yy}$ over the photoactivated area [Fig. 4G to H]. In the quiescent regime $\gamma_{xx}$ remained zero, indicating lack of any measurable motor-driven dynamics. Interestingly, $\gamma_{yy}$ decreased over time [Fig. 4H]. This can be attributed to the depletion-induced contraction (45). Indeed, never photoactivated flow-aligned samples exhibited slight contractions, pulling toward the chamber center [Fig. S11]. In the sliding regime, $\gamma_{xx}$ increased linearly with time, indicating a constant extension rate. Concurrently, $\gamma_{yy}$ decreased to the same extent as the quiescent regime, indicating lack of motor-driven transverse dynamics. In the buckling regime, $\gamma_{xx}$ grew quickly, increasing by more than 100% in 5 min. Simultaneously, $\gamma_{yy}$ became positive and grew rapidly, indicating filament motion perpendicular to the direction of extension.

We estimated the material flux through the boundary of the photoactivated region, by measuring the change in the average fluorescent intensity of labeled microtubules within the exposed region [Fig. 4I]. Similar to the strain analysis results, the fluorescent intensity in the quiescent regime showed a slight increase of material over time due to the depletion-induced contraction. In the sliding regime, the intensity decreased linearly as microtubules extended out of the photoactivated region along the *x*-axis. In the buckling regime, the intensity decreased rapidly as the bend instability pushed the microtubules out of the photo-activation region. When expelled in the background, microtubules became stationary and did not return to the exposed region. Thus, with continued illumination, the photo activated region eventually became devoid of microtubules [Video 6].

**Fig. 5.**
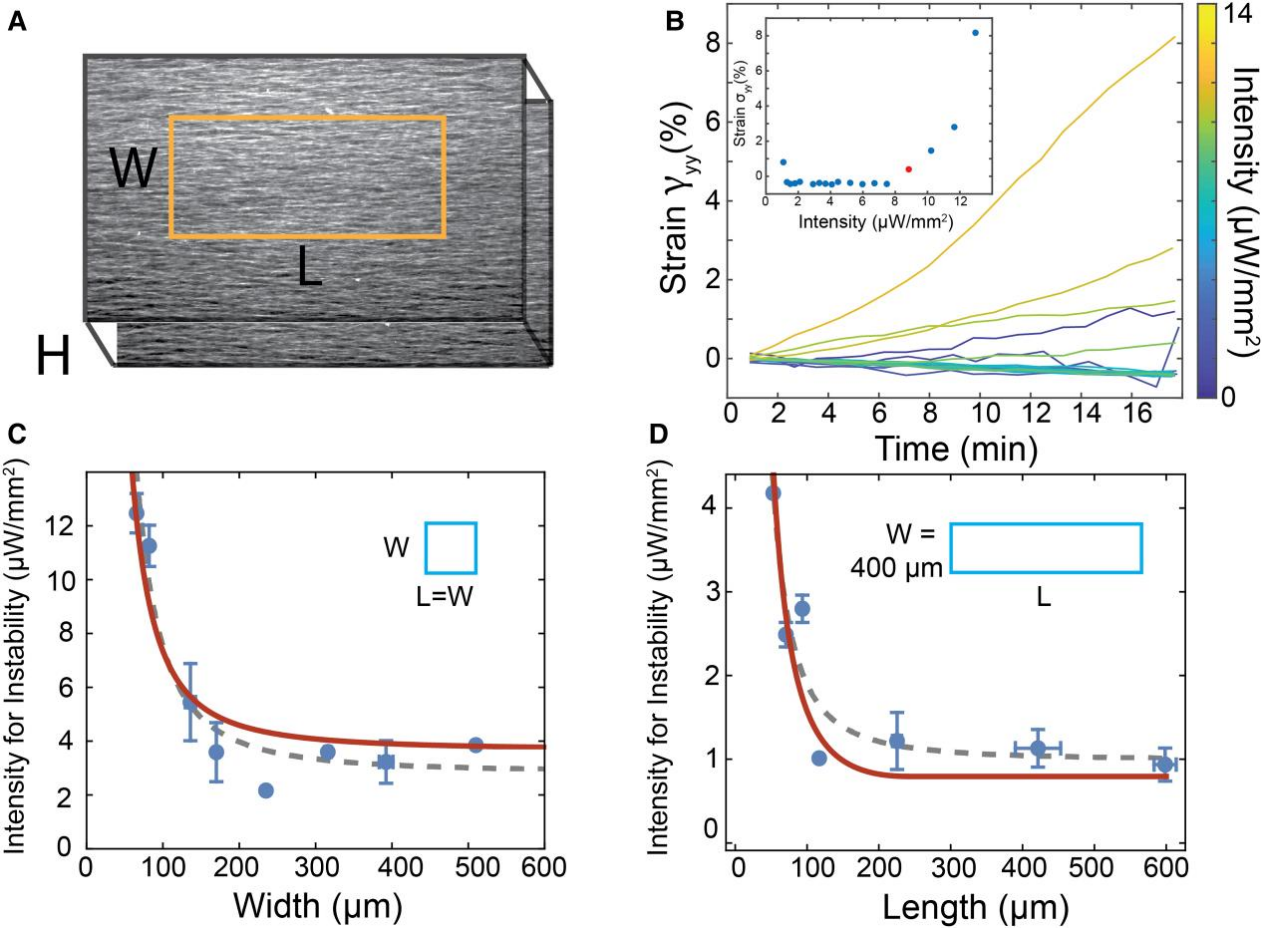
Length scale of the bend instability. A) A rectangular region (length *L* and width *W*) of flow-aligned microtubules is activated. The system confinement is three-dimensional, with the height *H* defined as the height of the chamber. B) $\gamma_{yy}$ plotted over time with increasing intensity (color). Inset: the strain after 17 min of photoactivation versus intensity. The bend instability is defined at the lowest intensity for which $\gamma_{yy}$ increases by more than 0.5% over 17 min, indicated by the red dot. C) The threshold intensity for instability plotted versus the L=W of a square activated region in a chamber H=100μm. Gray dashed line is fit to generic scaling of $1/L^{2}+C$. The solid red line is fit to theory Eq. 5 with a=1.17±0.1 nW${}^{-1}$. Error bars indicate standard error on multiple activated areas (n=2, 2, 4, 3, 1, 2, 3, 1 from W=50→500μm). D) The threshold intensity for instability for W=400μm plotted versus the length *L* of the activated region in a chamber H=300μm. Error bars indicate standard error on multiple activated areas (n=1, 2, 2, 1, 3, 2, 3 from L=50→600μm). Gray dashed line is fit to generic scaling of $1/L^{2}+C$. Solid red line is fit to theory Eq. 5 with a=0.93±0.06 nW${}^{-1}$.

The combination of the width *W* and the height *H* of the microfluidic channel determines the length scale of the bend instability (42). We illuminated aligned samples with rectangular patterns, with length *L* along the alignment direction and width *W* perpendicular to the alignment [Fig. 5A]. We systematically increased the light intensity to find the threshold activity required for the bend instability. The signature of bending is the growth of $\gamma_{yy}$. We defined a threshold intensity for the bend instability to be when the strain $\gamma_{yy}$ exceeded 0.5% over a 17 min activation [Fig. 5B, Fig. S12]. The intensity of light required to generate buckling in this time depended on the size of the illuminated region. For example, the intensity required to generate buckling of L∼100μm region was three times larger than for L∼500μm squares [Fig. 5C].

The threshold intensity for buckling exhibits a $\;1/L^{2}+C$ dependence on confinement length scale, where *C* is a fixed constant [Fig. 5C, Fig. S13]. The hydrodynamic theory predicts that the instability is controlled by a dimensionless activity parameter $\alpha_{\mathrm{eff}}=(\alpha (S_{0}+\xi ))/(2\eta D_{R}\kappa )$, where, $S_{0}$ is the magnitude of initial order, ξ is the flow alignment parameter, $D_{R}$ is the rotational diffusion, *κ* is the elastic constant, *η* is the viscosity of the active fluid [Supplementary material: Hydrodynamic Model]. The instability occurs when $\alpha_{\mathrm{eff}}$ is equal to a geometrical constraint such that:


(5) $$\alpha_{\mathrm{eff}}=\left\{\begin{array}{cc} \pi^{2}L^{2}{\left(\frac{1}{L^{2}}+\frac{1}{W^{2}}+\frac{1}{H^{2}}\right)}^{2}, & \frac{1}{L^{2}}>\frac{1}{H^{2}}+\frac{1}{W^{2}}\\ 4\pi^{2}\left(\frac{1}{W^{2}}+\frac{1}{H^{2}}\right), & \frac{1}{L^{2}}<\frac{1}{H^{2}}+\frac{1}{W^{2}} \end{array}\right\}$$

Here, *L*, *W*, and *H* are the dimensions of the activated region [Fig. 5A].

If we assume that the active stress is linearly proportional to the intensity of the optical signal, $I=a\alpha_{\mathrm{eff}}$, where *a* is a fit parameter, the hydrodynamic model captures the $1/L^{2}+C$ scaling of the experimental data [Fig. 5C]. The theory further predicts that if the confinement perpendicular to the direction of alignment is held constant W=400μm while the length *L* of the illuminated region varies, the threshold intensity will saturate when $1/L^{2}=1/W^{2}+1/H^{2}$ to an effective activity $4\pi^{2}(1/W^{2}+1/H^{2})$. Theoretical predictions agree with the experiments, where a=1.17±0.1 nW${}^{-1}$ and a=0.93±0.06 nW${}^{-1}$ for the respective fits [Eq. 5, Fig. 5C and D].

### Switching active stress from contracting to extensile

**Fig. 6.**
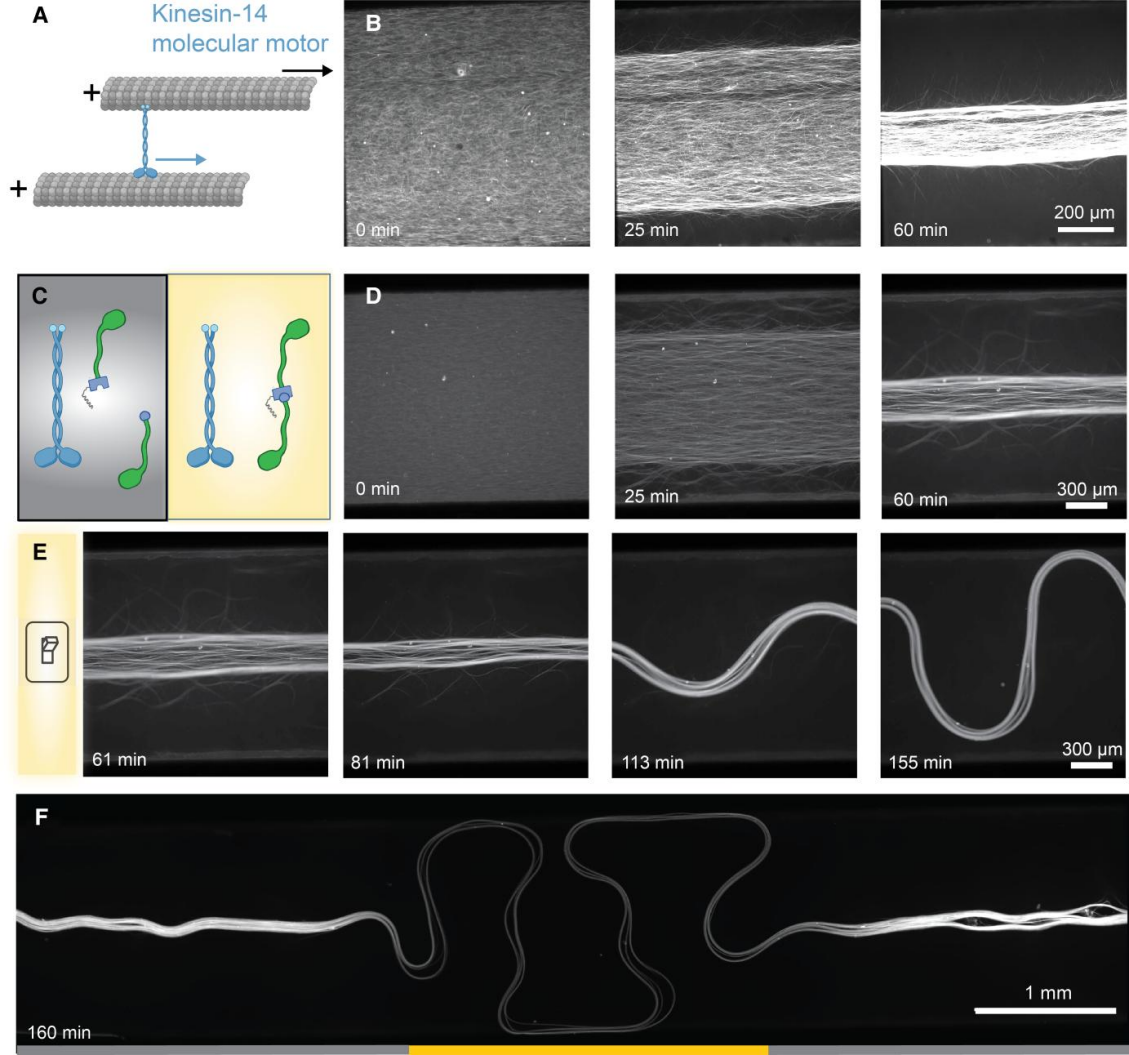
Optogenetic switch from contractile to extensile active stress. A) Kinesin-14 motor has a passive microtubule binding domain and a motor domain that steps toward the microtubule minus end (blue arrow). The kinesin drives interfilament sliding (black arrow). B) A time series of the kinesin-14-driven microtubule contractile dynamics of an aligned microtubule gel. C) Microtubule-based active matter powered by kinesin-14 and K365 opto-kinesin clusters. In the dark state, kinesin-14 dominates system dynamics, generating contraction. In the illuminated state, opto-kinesin clusters generate competing extensile stresses. D) A two motor-network with deactivated opto-kinesin clusters contracts. E) The contracting network is locally photoactivated with 10 μW/mm${}^{2}$ at 61 min, and the opto-kinesin induce extension. A time series shows the transition to extensile, buckling behavior. F) Entire sample after photo-activation at t=156 min. The yellow region has been illuminated. Outside this region, opto-kinesin remain deactivated.

So far, we demonstrated quantitative control of the extensile active stresses in space and time. In comparison, biological cells are able to control not only the magnitude but also the sign of the active stress to perform complex functions. Although both contraction and extension have been observed in a wide variety of cytoskeletal active matter systems (46–48), an on-demand switch between these two regimes has yet to be realized. Motivated by such reasoning, we explored the dynamics of a multi-motor material composed of K365 opto-kinesin clusters and full length kinesin-14. The latter is a minus-end directed molecular dimer motor with a passive microtubule-binding domain, a long neck linker, and a motor domain that steps processively at ∼20 nm/s, more than an order of magnitude slower than kinesin-1 [Fig. 6A] (49, 50). Thus, kinesin-14 can simultaneously passively bind one microtubule and advance towards the minus end of another microtubule, inducing their relative microtubule sliding (51). Kinesin-14 powers interfilament sliding in aligned microtubule networks that is independent of the local filament polarity (52).

In the presence of a depletion agent, kinesin-14 alone contracted a microtubule network [Supplementary material:Materials and Methods]. The active contraction led to the formation of a single macroscopic bundle aligned with the chamber walls, which continued contracting isotropically for tens of hours, pulling from the edges of the chamber along and perpendicular to the direction of alignment. [Fig. 6B, Video 7]. Next, we combined the kinesin-14 contracting network with K365 opto-kinesin [Fig. 6C]. When left in the dark, the material still formed a contracting network, as it would without the opto-kinesin [Fig. 6D]. However, upon illumination, the opto-kinesin clusters generated their own active stresses [Fig. 6E, Video 8]. The system exhibited extension and buckling in the photoactivated region which suggested extensile active stresses. The extensile behavior was localized to the activated region, thus the system simultaneously exhibited spatially distinct regions of extensile and contractile active stress [Fig. 6F]. On-demand transition from contractile to extensile stresses opens the door for new fundamental studies related to the sign of active stress, as well as engineering applications. However, the transition was not reversible, highlighting that the microscopic mechanism requires further study.

## Discussion

We developed and characterized optically responsive kinesin clusters of non-processive K365 motors, showing that they are optimized for controlling extensile active stresses. These motors add to a growing toolbox of optical control of active dynamics (22, 24, 25). The K365-opto clusters induced up to a 200-fold increase in the speed of autonomous flows between a dark and a fully illuminated state. Importantly, gels powered with K365-opto clusters displayed greater temporal regularity when compared to K401-opto clusters, a finding that parallels the analogous observation of conventional streptavidin-bound clusters (28). In comparison, the K401 opto-motors drove extensile active fluids for longer times and exhibited faster flows. These results have wide implications both within the field of active matter, and beyond.

Kinesin clusters can both generate active stresses and passively cross-link microtubule bundles (53–55). With decreasing ATP concentration, the motors’ primary role switches from generating active stresses to cross-linking filaments (53). Consequently, ATP controls a transition between a quiescent elastic solid and a spontaneously flowing active fluid. Opto-kinesin clusters provide a dynamical switch between an active fluid and a quiescent elastic network. In the dark state, the K365 opto-kinesin have a reduced tendency to dimerize. Thus, one might expect the microtubule network in the dark state to remain fluid-like. However, we found that upon deactivation, the network froze, and the bundles retained their structure. Upon re-activation, the flows were well correlated with the flow field measured just before deactivation [Fig. S14]. This behavior demonstrates, that even in the absence of motor clusters, a microtubule network is an elastic solid that is held together by PEG-induced depletion.

The properties of opto-kinesin provide insight into the bend instability, which is a ubiquitous feature of extensile fluids (39, 41, 42, 40). In conventional systems, a uniformly aligned state is unstable to spontaneously growing bend deformation that increase in time, eventually generating turbulent-like dynamics. Opto-kinesin clusters enable one to pause the bend generation. Once deactivated, the deformed network only partially relaxed, wherein the microtubule bundles locally straightened as if released from tension. In comparison, a deformed conventional liquid crystal, relaxes to a uniformly aligned state. The partial relaxation again demonstrates that microtubule-based fluids have complex viscoelastic and plastic properties, that remain unexplored.

Changing the photoactivation intensity of spatially illuminated shear-aligned sample, revealed a size-dependent lower critical illumination intensity, below which the bend instability was not observed. This is in agreement with theory, which predicts a suppression of the bend instability for active nematics that are confined below a critical length scale (39, 44). In comparison, the size-dependent suppression of the instability was not experimentally observed for shear aligned microtubule fluids confined in microfluidic channels (42). The notable difference between the two systems is the nature of the boundaries. Microfluidic channels aligned the filaments, but compressible isotropic active fluids could easily separate from the wall, leaving behind regions devoid of active material. In comparison, partially illuminated aligned gels remained physically connected to the background elastic network. When a confined region was activated below the instability threshold for long times, the region did eventually buckle [Video 9]. However, in this regime microtubules slid against each other, leading to net outflux of the material, suggesting that the material properties of the network changed, before the onset of the instability.

Light intensity controls the speed of the active flows. Two observations suggest a complex relationship between measured flow speeds and activity that warrants further studies. The active fluids exhibited a hysteresis in speeds [Fig. S15 A]. Increasing photoactivation intensity from low intensities increased the speed of the fluid flows. However, after saturating the system and returning to low intensities, the fluid speed was five times higher than the original photoactivation at the same intensity. In between photoactivation events, the dark speed remained constant and near zero, indicating that the motors returned to their low affinity dark state and exhibited no irreversible binding. There was a noticeable change in the gel structure between the early and late low-light activation [Fig. S15 B]. These data suggest that the network properties, rather than the motors contribute to the hysteresis. Furthermore, we also observed that the intensity for the speed saturation of the bulk fluid flow was lower than the intensities which induced sliding and buckling in the confined system. We attribute this to the hydrodynamic constraints of the system in which the activity (or intensity) required for saturation is inversely proportional to the confinement.

Our results have implications beyond the field of active matter. In one direction, control of extensile active stresses provide a foundation for experimentally steering turbulent dynamics towards a targeted dynamical state (56). These foundations also extend to attempts to control droplet motility in active liquid–liquid phase separation systems (57). In a different direction, in the absence of ATP, kinesin motors can bind to the microtubules, acting as photo-inducible cross-linkers that can be patterned in space and time to control the structure and mechanical properties of elastic fibrous networks (58).

## Supplementary Material

pgad130_Supplementary_Data

## Data Availability

Data is available from https://doi.org/10.5061/dryad.83bk3j9vh.

## Preprints

A preprint of this article is published at https://arxiv.org/abs/2209.06277.
