# Supplementary material for: Spatio-temporal patterning of extensile active stresses in microtubule-based active fluids
Source: PNAS Nexus. 2023 Apr 12;2(5):pgad130. doi: 10.1093/pnasnexus/pgad130 (PMC10165807; doi:10.1093/pnasnexus/pgad130)
Supplement: pgad130_Supplementary_Data [file pgad130_supplementary_data.zip › PNASNEXUS-PNASNEXUS-2022-00929-s10.pdf]

Spatiotemporal patterning of extensile active stresses in microtubule-based active fluids Supplementary Information

Linnea M. Lemma, Minu Varghese, Tyler D. Ross, Matt Thomson, Aparna Baskaran, Zvonimir Dogic

April 7, 2023

1 Experimental Methods

1.1 Protein Preparation

GMPCPP stabilized microtubules with an average length of 1 μm were prepared as previously described [1].The plasmids encoding for processive opto-kinesin K401 (K401-iLID: Addgene 122484 and K401-micro: Addgene 122485; gifts from Tyler Ross and Matt Thomson) were transformed into BL21 cells. After culturing for 4 hours in 2XYT media, expression was induced at OD 0.6 with IPTG and grown for 16 hours at 18°C. Cells were lysed using a Branson tip sonicator 550 with a 0.25 inch tip for 10 s at 30% power with 50 s recovery for 6 cycles. Lysate was then clarified by spinning at 100,000 RCF for 30 minutes. Purification was performed using an AKTA Fast Protein Liquid Chromatography system (GE Healthcare) with a 1 mL Nickel column (HisTrap, GE Healthcare) [1, 2]. The wash buffer used was 20 mM imidazole, 50 mM Sodium phosphate, 250 mM NaCl, 4 mM MgCl2, 0.05mM ATP, 5 mM βmercaptoethanol, 5% w/v glycerol, pH 7.2. The elution buffer was the same as the wash buffer but with 500 mM imidazole. For the K401-micro construct, the MBP tag was digested with TEV protease and removed by passing over a Nickel column. The buffer was exchanged into wash buffer and then diluted to 50% v/v glycerol. The kinesin motors were aliquoted, flash frozen in liquid nitrogen and stored at -80°C.

To construct the K365-iLID and K365-micro plasmids (Addgene 188456, 188457), we PCR amplified the desired regions [Fig. S3] from the K401-iLID plasmid (Addgene 122484, gift from Matt Thomson) and the K401-micro plasmid (Addgene 188457, gift from Matt Thomson) with Q5 High-Fidelity DNA polymerase (New England BioLabs). We then used HiFi DNA assembly kit (New England BioLabs) to circularize the product. We transformed the plasmids into BL21 cells, and expressed and purified the protein following the same protocol as the processive motors described above [2].

The active mixture was prepared without kinesin and microtubules at 1.4 mM ATP [1]. Briefly, an active mixture was prepared in M2B (80 mM PIPES, 2 mM MgCl2, 1 mM EGTA, pH 6.8) with a depletion agent to induce microtubule bundling, an ATP regeneration system to maintain activity and oxygen scavenging system to prevent photobleaching: 0.8% w/v PEG (35 kDa, Sigma Aldrich), 26.6 mM PEP (Beantown Chemicals product #129745), 1.4 mM ATP (Sigma Aldrich), 6.7 mg/mL glucose, 0.4 mg/mL glucose catalase, 0.08 mg/mL glucose oxidase, 5 mM $MgCl_2$. This active mixture was prepared once for all experiments with opto-kinesin. After mixing, the reagents were flash frozen in liquid nitrogen in 5 μL aliquots and stored at -80°C until the day of experiments.

1.2 Chamber Construction

Glass slides were treated with an acrylamide brush to prevent proteins sticking [3, 1]. The opto-kinesin constructs were especially sensitive to high coverage surface treatments, when compared to the conventional kinesin-streptavadin clusters. Parafilm was cut into chambers 1, 2 or 3 mm in width and sandwiched between two acrylamide slides. Using a hot plate, the parafilm sandwich was heated to 60°C. While on the hot plate, we gently pressed down on the parafilm spacer using the blunt end of an eppendorf 0.5 mL tube until the parafilm turned translucent. For experiments where the height of the chamber was altered, multiple parafilm spacers were carefully stacked on top of each other and the chamber was constructed as above.

1.3 Optical Microscopy

Images were taken on Nikon Ti2-Eclipse inverted microscope equipped with either a Photometrics Prime 95b or an Andor Zyla 5.5 sCMOS camera. Additionally, we used a Leica Resonant SP8 scanning confocal with Stellaris white light laser for some confined activation and subsequent imaging.

1.4 Sample Preparation

All the samples were prepared in conditions where the sample was only exposed only to red light from an red LED-equipped desk lamp. Microtubules, active mixture aliquots and kinesin motors were rapidly thawed. Opto-kinesin motor constructs were added to 300 nM concentration. This was the minimal concentration at which we reliably obtained extensile active gels while photoactivated. Higher concentrations of motors increased the dark state velocities and decreased the sample lifetime. Components were mixed for final desired concentrations and immediately loaded into chamber. The chamber was sealed using a fast setting silicone polymer (Picodent Twinsil Speed).

1.5 Sample Preparation of Multimotor Composite

The multimotor composite was prepared in an active mixture at 80 mM PIPES, 5 mM magnesium chloride, 1 mM EGTA, 0.034% pyruvate kinase, 52 mM PEP, 1.4 mM ATP, 0.5 mg/mL tubulin and 0.1% PEG. An oxygen scavenging system was also added to prevent photobleaching (6.7 mg/mL glucose, 0.4 mg/mL glucose catalase, 0.08 mg/mL glucose oxidase). The final concentrations of K365 opto-kinesin was 450 nM. The final concentration of kinesin-14 was 125 nM. The sample was photoactivated using Lumencor Sola light engine through a 472 ±30 nm excitation filter at intensity of 10 μW/mm^2.

1.6 Patterning active stress

For bulk activation experiments, the epi-fluorescence arm of the microscope was used to illuminate the sample with blue light (Lumencor, Sola light engine white LED, 472 ±30 nm excitation filter). Initially, the entire chamber was exposed to 37 mW/mm^2 of continuous blue light using a Lumencor Sola light engine through a 1× 0.04 NA objective (Nikon Instruments, CFI Plan Achromat 1X) for 10 minutes. This allowed the sample to obtain its isotropic bundled structure. Subsequent experiments were performed at 4× 0.13 NA (Nikon Instruments, CFI Plan Fluor 4X) or 4× 0.2 NA (Nikon Instruments, CFI Plan Apochromat Lambda D 4X) with pulsed blue light.

For spatial patterning of activity, we used a laser scanning confocal (Leica Microsystems, SP8) to the activate regions of arbitrary shape with a laser tuned to 488 nm (Leica Microsystems, Stellaris White Light Laser). Alternatively, an opaque mask was placed above the sample and a white LED (Nikon Instruments, Dia LED) was used to photoactivate the exposed region from above. The data in Fig. 5 was taken using opaque masks. For all experiments, the integrated intensity was calculated by first, measuring the power at the sample plane with a power meter. The entire sensor was filled by light, so that the intensity of light $I_{\text{raw}} = \frac{\text{power}}{\text{area of sensor}}$. To account for the pulsed activation,

$$I_{\text{integrated}} = I_{\text{raw}} \times \frac{\text{exposure time}}{\text{frame interval}}. \quad (1)$$

This assumption is valid only within the regime where the motors are not unbinding on the timescales of the frame interval. We found this threshold to be 30 s from an experiment in which we incrementally increased the time between photoactivation pulses.

1.7 Analysis of Microtubule Flows

Flow fields were obtained through particle image velocimetry (PIVLab MATLAB plugin) [4]. The PIV analysis parameters were optimized for each data set [Fig. S1]. Generally, a small window size in pixels resulted in larger average speeds. Thus, we kept the window size constant across data sets. By incrementally skipping frames in the analysis, we determined the optimal sampling

of data to suppress noise and maintain temporal and spatial resolution. The average speed for each frame was calculated within the center of the chamber.

For Fig. 2, the average speeds were obtained from imaging samples prepared as described above for 2-6 hours during which they were continuously photoactivated. The average was taken over the entire imaging time.

1.8 Analysis of Network Relaxation

For Fig. 3, the average orientation of the network was found from the structure tensor of the fluorescent image of the microtubules using the FIJI Image J plugin OrientationJ with a window size of 8 pixels [5]. The initial angle was set to zero radians, defined to be along the x axis. The standard deviation reported in Fig. 3(b) is from the spatially resolved orientation at a particular time.

1.9 Calculation of strain fields

The displacement and strain fields were calculated from the fluorescent images of the microtubules using NCORR2 in MATLAB [6]. The transition to instability was defined by the in-plane component of the strain perpendicular to the network alignment, γ_{yy}. Over the 17 minute activation for each intensity, we defined the onset of buckling as when the strain increase beyond 0.5%. Below this threshold, the strain fluctuated around the noise floor [Fig. S11].

2 Elastic Relaxation of the bend instability

We measured the average angle of a flow aligned microtubule network driven by non-processive opto-kinesin. Upon deactivation, the angle partially relaxed towards a more uniform state. We describe a model that accounts for the lingering activity of motors that are being deactivated over time and the elastic decay of the microtubule network.

A function $M(t)$ describes the fraction of unbound motors where $t = 0$ is the last moment of photoactivation. Thus, $M(t = 0) = 0$ indicates that the bound motor fraction is completely saturated. In the dark, we will assume that $M(t \to \infty) = 1$ indicating that the unbound motor fraction is completely saturated. $M(t)$ should take on a form that is consistent with the unbinding kinetics of proteins. We define

$$M(t) = 1 - e^{-t/\tau}. \quad (2)$$

Absorbance assay revealed the thermal reversion kinetics of the iLID domain is exponential decaying with characteristic time scale $\tau = 24$ s assuming exponential kinetics [Fig. S8] [7] . It follows that the angle relaxation is described by

$$\langle\theta\rangle = (1 - M(t))A(t) + R(t) \quad (3)$$

$$\langle\theta\rangle = e^{-t/\tau}A(t) + R(t) \quad (4)$$

where $A(t)$ is the activity-driven growth of the angle and $R(t)$ is the elastic relaxation of the network. We measured $A(t)$ during the activation cycle and found $A(t) = \gamma t$, where $\gamma = 0.002$ rad/s [Fig. S7].

We assume that the microtubule network behaves as a Hookean elastic solid such that its relaxation can be described by an overdamped spring

$$\ddot{x} + \beta\dot{x} + \omega_o x = 0 \tag{5}$$

where the solution is given by the ideal limit conditions

$$R(t) = C * e^{-t/r} \tag{6}$$

where $r = 2\left(-\beta - \sqrt{\beta^2 - 4\omega_o^2}\right)^{-1}$ where β describes the frictional dissipation and ω_o is the angular frequency of oscillation.

Putting this together, we fitted the measured decay curves to:

$$\langle\theta\rangle = e^{-t/\tau}\dot{\gamma}t + e^{-t/r} \tag{7}$$

where r is a fit parameter describing the characteristic time for the solid's relaxation. To compare relaxations at different stages of the bend instability we normalize the angle $\langle\theta_{\mathrm{norm}}\rangle = \frac{\theta_{\mathrm{i}} - \theta_{\mathrm{min}}}{\theta_{\mathrm{max}} - \theta_{\mathrm{min}}}$.

3 Hydrodynamic Model of Confined Aligned Microtubule Network

We consider a flow aligned active nematic with spatially patterned active stresses. In the illuminated region, the extensile active stresses destabilize orientational order. Let the instantaneous axis of orientational order of the microtubule bundles at position $\vec{r}$ be denoted $\hat{n}(\vec{r}, t)$. $\hat{x}$ is the axis of initial nematic order, i.e., $\hat{n}(t = 0) = \hat{x}$. Consider a spatially varying perturbation to the orientationally ordered state, such that $\hat{n} = \hat{x} + \delta\vec{n}_\perp(\vec{r})$. Modelling the illuminated region using the hydrodynamic theory for an active nematic reveals that the eigenvectors of the dynamics correspond to Fourier transforms of twist-bend and splay-bend deformations, and that twist-bend modes grow faster than splay-bend modes [8]. Therefore, the instability seen in the experiment should correspond to a growing twist-bend deformation. Further, the dynamics of a twist-bend deformation with wavevector $\vec{k}$ is given by (see SI of Chandrakar et. al., 2020 [8] for the hydrodynamic model and the linear stability analysis)

$$\partial_t[\hat{x}.(\vec{k} \times \delta\tilde{\vec{n}}_\perp)] = \left(-D_R\kappa k^2 + \frac{2\alpha}{3\eta}\frac{k_x^2}{k^2}\right)[\hat{x}.(\vec{k} \times \delta\tilde{\vec{n}}_\perp)] \tag{8}$$

where α is the "activity" which corresponds to the force dipole generated by the extending microtubule bundles, D_R is the rotational diffusion constant of the microtubule bundles, κ is the nematic elasticity (assuming that bend, twist, and splay, all have the same energetic cost), and η is the viscosity. Let H be the

height of the chamber, L be the size of the illuminated region along $\hat{x}$, and W be the size of the illuminated region along $\hat{y}$. Then, the components of the wave vector are quantized by the confinement dimension, and are restricted to be such that $k_x > \pi/L, k_y > \pi/W,\ k_z > \pi/H$. Based on experimental observations, we assume $k_y = \pi/W$ and $k_z = \pi/H$. Then, depending on the value of L, there are two instability regimes:

1. At large L, the instability sets in as soon as the activity is high enough for the fastest growing wave mode from equation 8 to be unstable. Taking the derivative of the growth rate in equation 8 with respect to k_x and equating it to zero, the fastest growing mode satisfies

$$\frac{\alpha(S_0+\xi)}{2\eta D_R\kappa} = \frac{(k_x^2+(\pi/W)^2+(\pi/H)^2)^2}{(\pi/W)^2+(\pi/H)^2} \tag{9}$$

and is unstable (equation 8 with this value of k_x is positive) only for

$$\frac{\alpha(S_0+\xi)}{2\eta D_R\kappa} > 4((\pi/W)^2+(\pi/H)^2) \tag{10}$$

From eqns 9 and 10, the fastest growing mode, if unstable has to satisfy

$$k_x^2 > (\pi/W)^2+(\pi/H)^2 \tag{11}$$

If L is large enough that $(1/L)^2 < (1/W)^2 + (1/H^2)$, equation 11 implies that $k_x > \pi/L$, so the fastest growing wave mode is not disallowed by confinement in the x dimension. Then, the threshold activity for the instability is given by equation 10. To summarize, for large L (i.e., $(1/L)^2 < (1/W)^2 + (1/H^2)$), the activity threshold for the instability is given by $\frac{\alpha(S_0+\xi)}{2\eta D_R\kappa} > 4\pi^2\left(\frac{1}{W^2}+\frac{1}{H^2}\right)$

2. For small L ($\frac{1}{L^2} > \frac{1}{W^2}+\frac{1}{H^2}$), even when the activity is high enough for the emergent fastest growing mode given by equation 9 to be unstable, there is no instability because this emergent mode is disallowed by the boundaries. Instead, the instability is observed when the growth rate of the $k_x = \frac{\pi}{L}$ mode becomes positive, i.e. (from eq. 8),

$$\frac{\alpha(S_0+\xi)}{2\eta D_R\kappa} > \pi^2 L^2\left(\frac{1}{L^2}+\frac{1}{W^2}+\frac{1}{H^2}\right)^2 \tag{12}$$

4 Supplementary Figures

Figure S1: PIV flow fields from fluorescence images.
Figure S2: Reduced difference between on and off flows in active gels driven by processive opto-kinesin upon repeated photoactivation.
Figure S3: Kinesin motor coding regions.
Figure S4: In the dark, the measured microtubule flows for the K365 opto kinesin are zero.
Figure S5: Average flow speed of extensile gel versus activation intensity.
Figure S6: Measuring network angle from microtubule fluorescence using OrientationJ.
Figure S7: Measuring the angular growth rate from the onset of the bend instability.
Figure S8: Extracting reversion timescale from absorbance recovery of iLID construct.
Figure S9: Fitting the microtubule network decay to the elastic model.
Figure S10: Displacement and strain fields of spatially patterned active stress.
Figure S11: Depletion induced contraction of a flow aligned sample.
Figure S12: Defining threshold intensity for instability.
Figure S13: Threshold intensity for buckling versus confinement.
Figure S14: Flow fields after de-activation are highly correlated with pre-activation flows.
Figure S15: Hysteresis in intensity-controlled active flow speed.

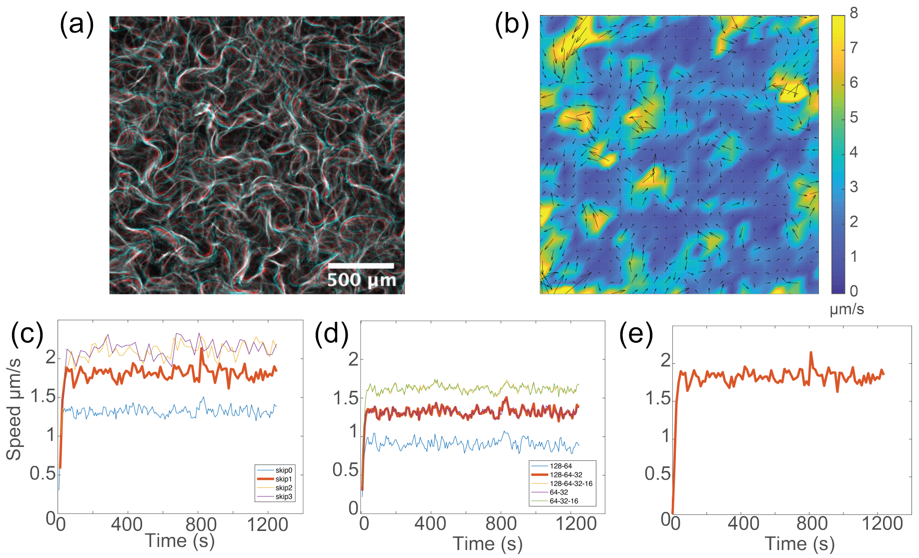

Figure S1: PIV flow fields from fluorescent microtubule images. (a) Overlay of two images taken at t=0 (red) and t=5 s (cyan) illustrate the dynamical microtubule network. **(b)** Flow field of images in (a) where colormap shows speed. **(c)** Average speed versus time for various PIV window sizes. **(d)** Average speed versus increasing frame interval. Parameters used for this data set are represented by bolded curve. **(e)** The chosen optimal mean speed versus time curve for this data set.

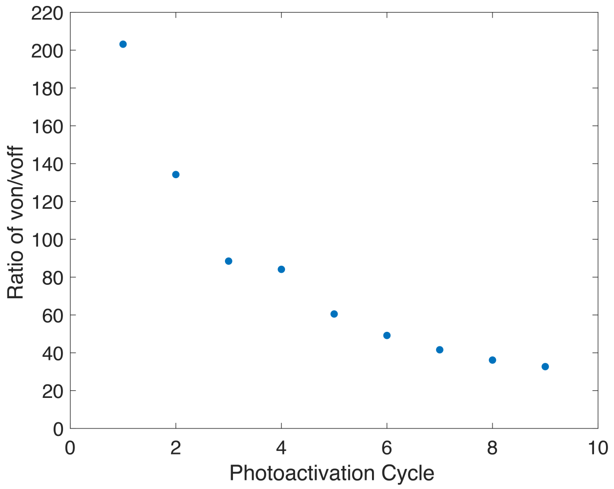

Figure S2: Aging of active gels powered with K401 opto-kinesin clusters. The ratio of microtubule flow speeds in the illuminated and dark state as a function of the photoactivation cycle.

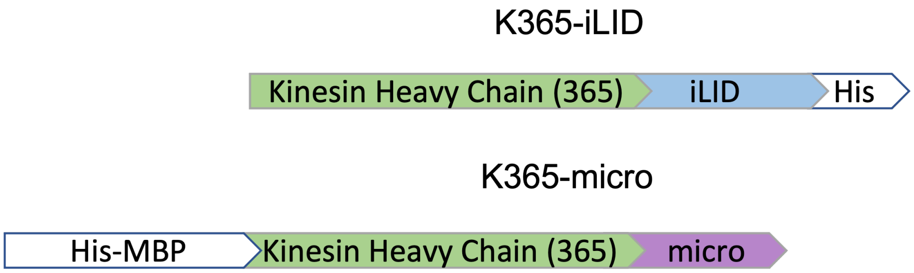

Figure S3: Kinesin motor coding regions

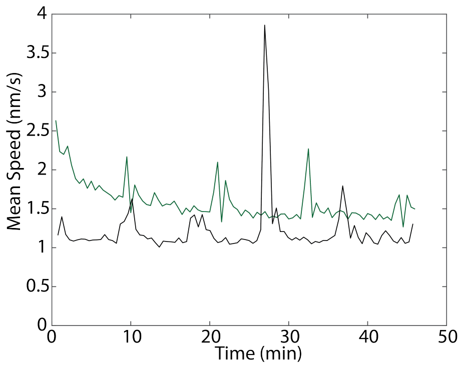

Figure S4: Dark-state dynamics of K365 opto-kinesin active fluids. The velocity of active fluid where the sample is in the dark (green) and a sample that ran out of ATP (black). Both analyses were done at 30 s/frame and the same PIV settings.

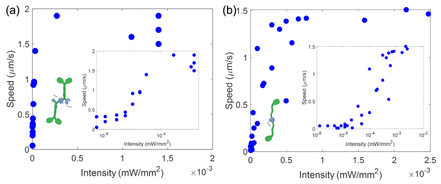

Figure S5: Average flow speed of extensile gel versus activation intensity. (a) A scatter plot showing individual experiments' measured flow speed for K401-opto driven extensile gels. (b) A scatter plot showing individual experiments' measured flow speed for K365-opto driven extensile gels. These data were binned and averaged to generate Fig. 2 in the main text.

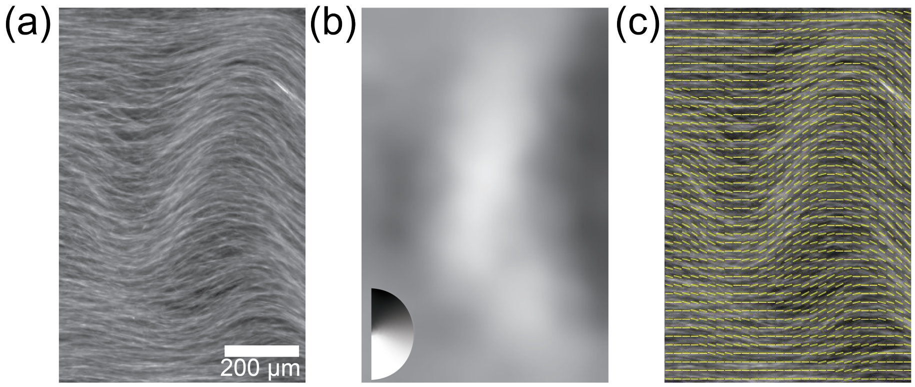

Figure S6: Measuring network angle from microtubule fluorescence using OrientationJ. (a) Fluorescence image of shear-aligned microtubules undergoing bend instability. (b) Filament orientation obtained from the structure tensor analysis using OrientationJ with window size of 8 pixels. Intensity indicates angle. (c) Calculated orientation field plotted over fluorescence image.

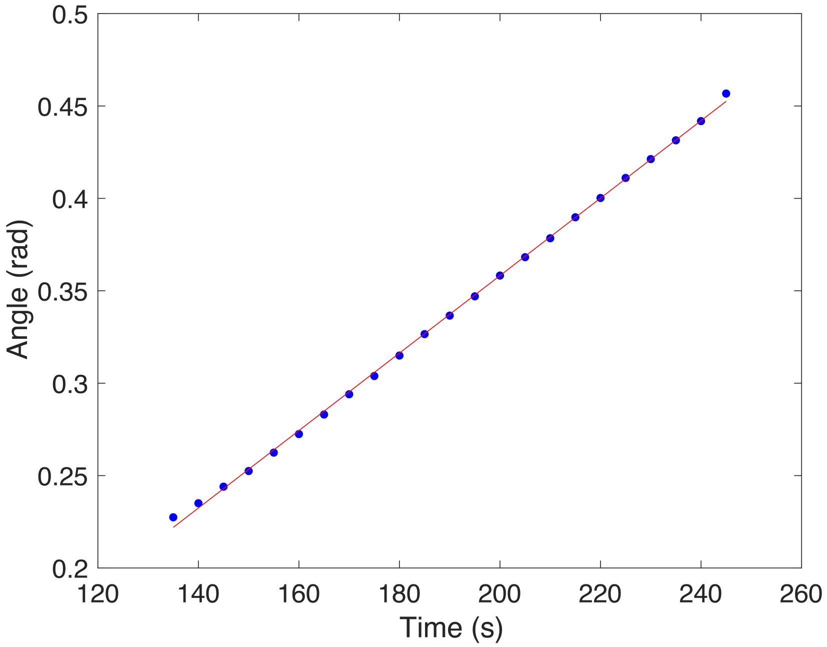

Figure S7: Measuring angular growth rate from the onset of the bend instability. The filament orientation angle versus time during activation of the bend instability. The line is a fit with the slope $\gamma = 0.002$ rad/s.

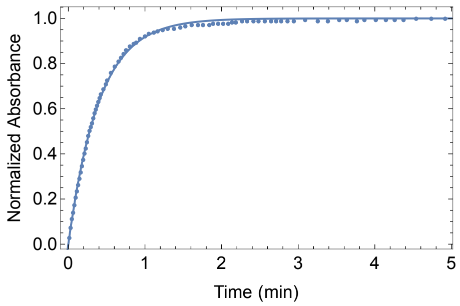

Figure S8: Extracting reversion timescale from absorbance recovery of iLID construct. Modified from [7]. A plot of the absorbance at 450 nm recovery after activation for the iLID construct where an absorbance of 1 indicates a full recovery to the dark structure of the iLID protein. The line indicates a fit to a bounded exponential $A = 1 - e^{-t/\tau}$ where $\tau = 24$ s.

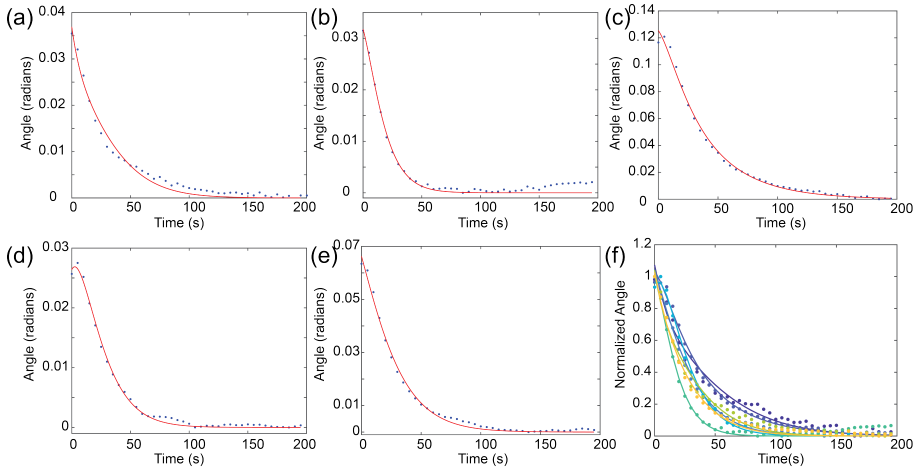

Figure S9: Fitting the microtubule network decay to the elastic model. (a-e) After de-activation a sample undergoing bend-instability undergoes partial relaxation. Fits of average angle $\langle\theta\rangle$ to Equation 1 after deactivation. (f) Data and fits were normalized by the maximum angle such that $\langle\theta_{\mathrm{norm}}\rangle = \frac{\langle\theta\rangle - \theta_{\mathrm{min}}}{\theta_{\mathrm{max}} - \theta_{\mathrm{min}}}$. The average is shown in Fig. 3(b).

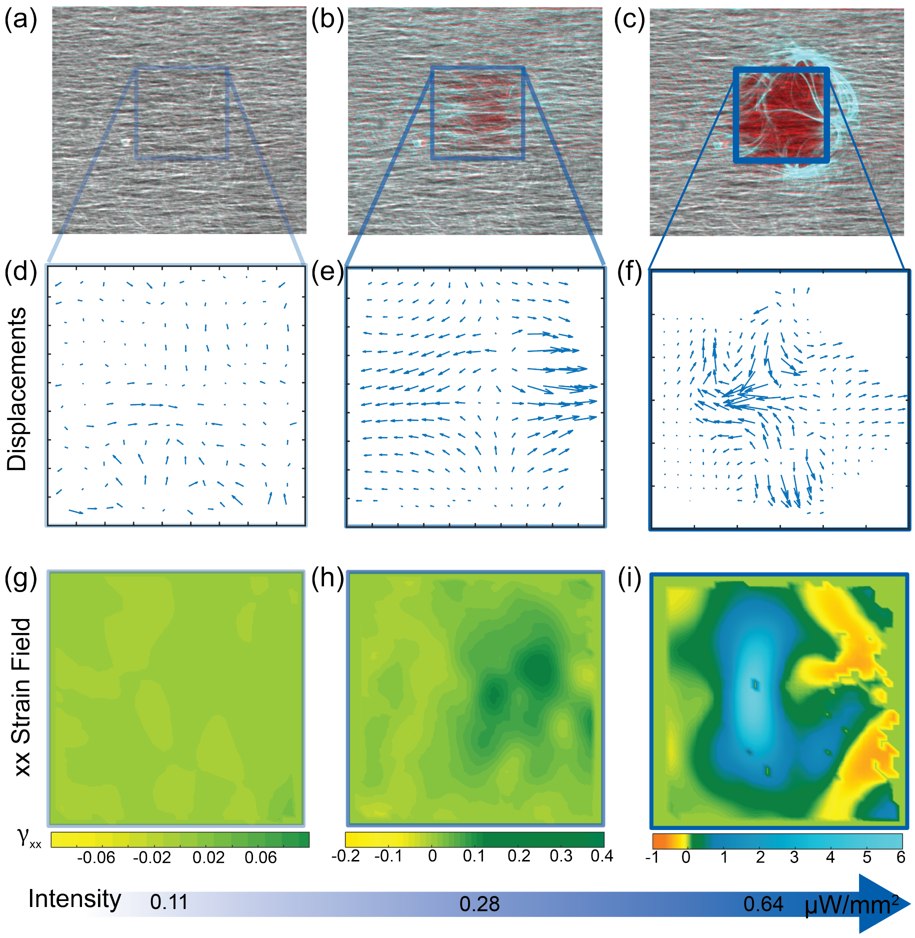

Figure S10: Displacement and strain fields of spatially patterned active stress. (a-c) Overlay of initial (red) and final (cyan) of a flow aligned 3-dimensional network of microtubules where the highlighted region is activated with light. Gray indicates no movement of filaments (red + cyan = white). Indicated squares are $400 \times 400\ \mu m^2$. **(a)** Quiescent regime: the intensity in the activated region, indicated by the yellow square, is low $0.11 \mu W/mm^2$. **(b)** Sliding regime: the intensity in the activated region, indicated by the green square, is $0.28\ \mu W/mm^2$. **(c)** Turbulent regime: the intensity in the activated region, indicated by the blue square is high $0.64\ \mu W/mm^2$. **(d-f)** Displacement field of the activated regions in (a-c) respectively. **(g-i)** Strain field γ_{xx} measured from the displacements in (d-f) respectively.

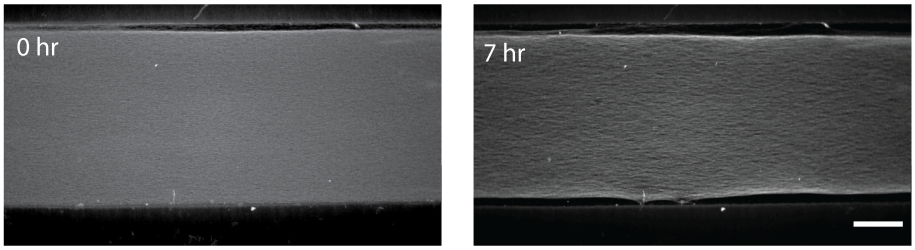

Figure S11: Depletion induced contraction of a flow aligned sample. Snapshots of an aligned microtubule network exhibiting depletion induced contraction.

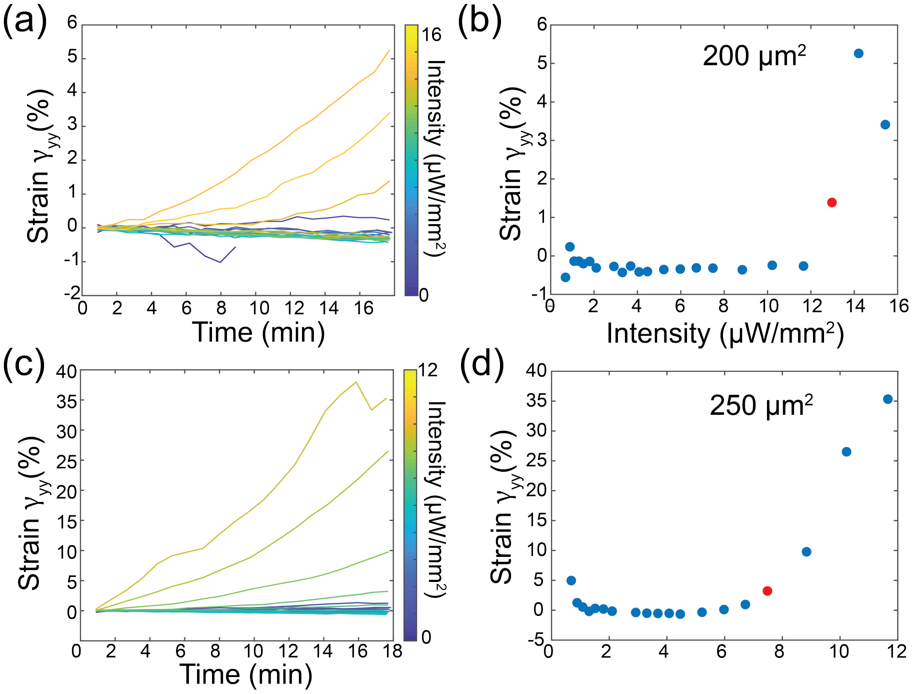

Figure S12: Defining threshold intensity for instability. (a) The component of strain perpendicular to alignment γ_{yy} as function of time for a 200 μm^2 activated region. Color indicates the intensity of activation light at 488 nm. **b** The strain γ_{yy} accumulated after 17 min of activation as a function of activation intensity for a 200 μm^2 activated area. The red data point indicates the intensity threshold for instability. **(c-d)** The same measurements for a 300 μm^2 activation region. The red data point indicates the threshold intensity for the instability.

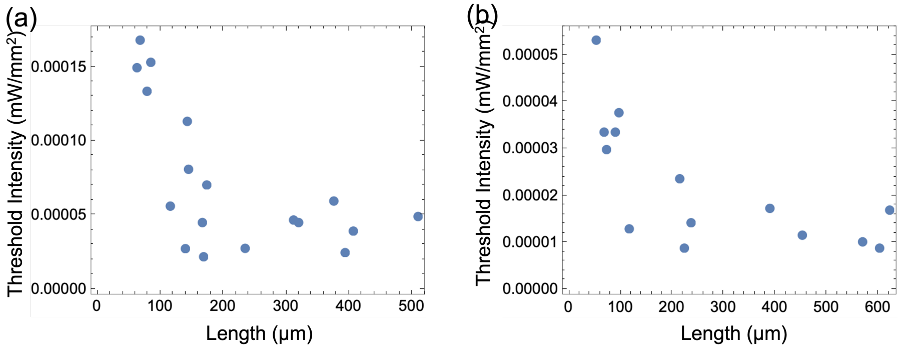

Figure S13: Threshold intensity for buckling versus confinement. (a) A scatter plot showing each measured threshold intensity for buckling for $L = W$ and $H = 100$ μm. (b) A scatter plot showing each measured threshold intensity for buckling for $L = 400$ μm and $H = 300$ μm. These data were binned and averaged for Fig. 5 in the main text.

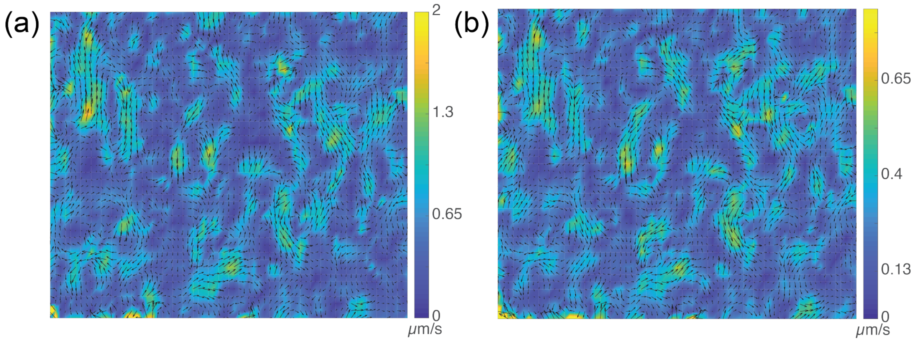

Figure S14: Flow fields after de-activation are highly correlated with pre-activation flows. (a) Flow field in last frame of of a light cycle PIV. Color indicates flow speed. (b) Re-activated flow field after 30 min dark cycle.

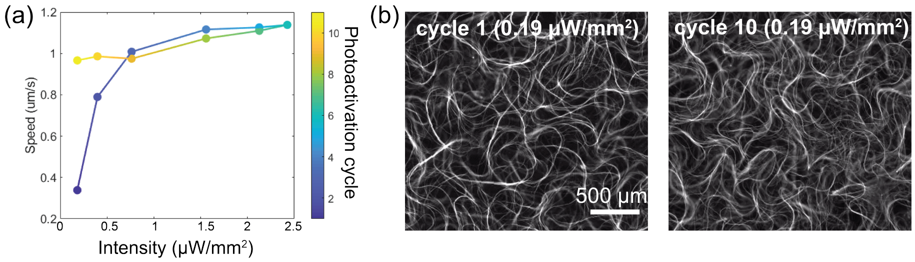

Figure S15: Hysteresis in intensity-controlled active flow speed. (a) The flow speed plotted as a function of photoactivation intensity. The color bar indicates forward in time from blue to yellow. **(b)** Bundle structure at the same photoactivation intensity for the cycle 1 and cycle 10 respectively.

5 Video Captions

Video 1: K401 opto-kinesin drive extensile active fluid dynamics when photoactivated. This video shows fluorescently labeled microtubules in an active fluid alternately photoactivated by 2.4 μW/mm^2 blue light and deactivated. Scale bar: 500 μm.

Video 2: K365 opto-kinesin drive extensile active fluid dynamics. This video shows fluorescently labeled microtubules in an active fluid alternately photoactivated by 2.2 μW/mm^2 blue light and deactivated. Scale bar: 500 μm.

Video 3: Controlling the onset of the bend instability. An initially aligned microtubule network with 300 nM K365 opto-kinesin was exposed to bursts of photoactivation which pushed the microtubules into the bend instability. Subsequent deactivation showed relaxation of the passive microtubule network as the K365 opto-kinesin un-clustered.

Video 4: Size dependence of bend instability. An initially aligned network of fluorescently labeled microtubules and K365 opto-kinesin was photoactivated inside the yellow boxes of various sizes (50 μm^2 - 500 μm^2). At a single photoactivation intensity, the largest box (500 μm^2) underwent the bend instability, the mid-size boxes (250 μm^2, 200 μm^2) mostly exhibited sliding dynamics and the smaller boxes (100 μm^2 and 50 μm^2) remained quiescent.

Video 5: Confined flows in aligned microtubule network for quiescent, sliding and bend instability regimes. An initially aligned network of fluorescently labeled microtubules was sequentially photoactivated at increasing intensities within a 400 μm^2 box indicated by the yellow border. Left panel: quiescent regime at low activation intensity, 0.11 μW/mm^2. Middle panel: sliding regime at 0.28 μW/mm^2. Right panel: bend instability regime at high activation intensity, 0.64 μW/mm^2.

Video 6: Depletion of microtubules within activated region. An initially aligned network of fluorescently labeled microtubules and K365 opto-kinesin with a photobleached line as a material marker. The region inside the yellow box was photoactivated with a 488 nm laser. The microtubules undergo successive bend instabilities and eventually deplete from the activated region.

Video 7: Kinesin-14 and K365 opto-kinesin without activation contracts for many hours. An active network of fluorescently labeled microtubules with both kinesin-14 and K365 opto-kinesin motors. The sample was never photoactivated so that the K365 opto-kinesin were un-clustered. The network contracts for the acquisition time, 12 hours.

Video 8: Transition from contractile to extensile active stress in composite motor system. An active network of fluorescently labeled microtubules with kinesin-14 motors and K365 opto-kinesin motors. Initially the K365 opto-kinesin were not activated, so that the network contracted into a dense aligned bundle driven by kinesin-14 motors. Upon photoactivation, the material extended and underwent the bend instability.

Video 9: Extended illumination below "threshold" intensity within confined region eventually leads to the bend instability. An initially aligned microtubule network is exposed to low intensity photoactivation light

within the indicated 400 μm^2 square. Eventually, the microtubules slide out of the activated region and the material bends perpendicular to the direction of alignment.

References

[1] Alexandra M Tayar, Linnea M Lemma, and Zvonimir Dogic. Assembling microtubule-based active matter. In *Microtubules*, pages 151–183. Springer, 2022.

[2] Tyler D. Ross, Heun Jin Lee, Zijie Qu, Rachel A. Banks, Rob Phillips, and Matt Thomson. Controlling organization and forces in active matter through optically defined boundaries. *Nature*, 572(7768):224–229, 2019.

[3] A. W. C. Lau, A. Prasad, and Z. Dogic. Condensation of isolated semi-flexible filaments driven by depletion interactions. *EPL (Europhysics Letters)*, 87(4):48006, aug 2009.

[4] William Thielicke and Eize Stamhuis. Pivlab–towards user-friendly, affordable and accurate digital particle image velocimetry in matlab. *Journal of open research software*, 2(1), 2014.

[5] Zsuzsanna Püspöki, Martin Storath, Daniel Sage, and Michael Unser. *Transforms and Operators for Directional Bioimage Analysis: A Survey*, pages 69–93. Springer International Publishing, 2016.

[6] J. Blaber, B. Adair, and A. Antoniou. Ncorr: Open-source 2d digital image correlation matlab software. *Experimental Mechanics*, 55(6):1105–1122, 2015.

[7] Ryan A. Hallett, Seth P. Zimmerman, Hayretin Yumerefendi, James E. Bear, and Brian Kuhlman. Correlating in vitro and in vivo activities of light-inducible dimers: A cellular optogenetics guide. *ACS Synthetic Biology*, 5(1):53–64, 01 2016.

[8] Pooja Chandrakar, Minu Varghese, S.Ali Aghvami, Aparna Baskaran, Zvonimir Dogic, and Guillaume Duclos. Confinement controls the bend instability of three-dimensional active liquid crystals. *Phys. Rev. Lett.*, 125:257801, Dec 2020.
